# Referral of children seeking care at private health facilities in Uganda

**DOI:** 10.1186/s12936-017-1723-1

**Published:** 2017-02-14

**Authors:** Anthony K. Mbonye, Esther Buregyeya, Elizeus Rutebemberwa, Siân E. Clarke, Sham Lal, Kristian S. Hansen, Pascal Magnussen, Philip LaRussa

**Affiliations:** 1 Ministry of Health, Box 7272, Directorate of Clinical and Community Services, Kampala & Department of Community & Behavioural Sciences, School of Public Health, Makerere University, Kampala, Uganda; 2 Department of Disease Control and Environmental Health, School of Public Health, Makerere University, Kampala, Uganda; 3 Department of Health Policy, Planning and Management, School of Public Health, Makerere University, Kampala, Uganda; 4 Department of Disease Control, London School of Hygiene and Tropical Medicine, Keppel Street, London, WC1E 7HT UK; 5 Department of Global Health and Development, London School of Hygiene and Tropical Medicine, 15-17 Tavistock Place, London, WC1H 9SH UK; 6 Institute for International Health, Immunology and Microbiology, Centre for Medical Parasitology and Institute for Veterinary Disease Biology, Faculty of Health and Medical Sciences, University of Copenhagen, Copenhagen, Denmark; 7 Department of Paediatrics, College of Physicians & Surgeons, Columbia University, New York, USA

## Abstract

**Background:**

In Uganda, referral of sick children seeking care at public health facilities is poor and widely reported. However, studies focusing on the private health sector are scanty. The main objective of this study was to assess referral practices for sick children seeking care at private health facilities in order to explore ways of improving treatment and referral of sick children in this sector.

**Methods:**

A survey was conducted from August to October 2014 in Mukono district, central Uganda. Data was collected using a structured questionnaire supplemented by Focus Group Discussions and Key Informant interviews with private providers and community members.

**Results:**

A total of 241 private health facilities were surveyed; 170 (70.5%) were registered drug shops, 59 (24.5%) private clinics and 12 (5.0%) pharmacies. Overall, 104/241 (43.2%) of the private health facilities reported that they had referred sick children to higher levels of care in the two weeks prior to the survey. The main constraints to follow referral advice as perceived by caretakers were: not appreciating the importance of referral, gender-related decision-making and negotiations at household level, poor quality of care at referral facilities, inadequate finances at household level; while the perception that referral leads to loss of prestige and profit was a major constraint to private providers.

**Conclusion:**

In conclusion, the results show that referral of sick children at private health facilities faces many challenges at provider, caretaker, household and community levels. Thus, interventions to address constraints to referral of sick children are urgently needed.

## Background

Although Uganda achieved the Millennium Development Goal target on child health (MDG4), there is continued effort to improve childhood morbidity and mortality [1]. It has been shown that delayed treatment-seeking, inappropriate treatment and poor referral to higher levels of care of sick children that seek care from health facilities are amongst some of the contributory factors [2, 3]. Studies in Uganda and elsewhere have found out that referral of sick children to higher levels of care is poor [3–6]. A another study in Uganda found that of the 70% of patients who sought treatment at private clinics within 1 week of onset of symptoms only 7% were properly managed (treated according to national guidelines), [3]. Several studies have estimated referral of children to be as low as 8% [6, 7].

In general, studies have found that referral of sick children to higher levels of care is poor, irrespective of whether a child is initially referred by a community health worker, a drug shop vendor or a government health facility [7, 8].

Several factors have been attributed to the poor referral of sick children like long distances to health facilities, high costs involved in referral, poor attitudes of health workers, lack of drugs at health facilities and lack of involvement of fathers in the referral process [9]. Referral is both complex and context-specific, with decision making about whether to make or take up a referral unfolding in different contexts (pharmacies; private clinics and shops; public clinics; and within households [10–12]. The decision by a health provider to refer patients to a higher level of services is contingent on clinical judgment, prior referral experience, perceived priorities, abilities of patients, caregivers ‘perceived implications for future patients and relationships with those referred.

Decisions to refer and take up referral are driven by different social actors (healthcare providers, household members and individual caregiver/patients); and are made in reference to confidence in diagnosis and desire to get better efficacy, cost, distance to the referral clinic, time and transport and perceptions about quality of care and availability of drugs at the receiving clinic [3, 13]. Recognizing the household as a major decision making forum regarding referral has brought into focus the importance of understanding household negotiations and influence of gender relations on the ability of patients/caregivers to secure resources and/or consent to take up a referral, cost being a major issue [6, 7, 14].

Studies of treatment-seeking behaviour in sub-Saharan Africa show that 50% of those with febrile illness access care through retailers, and that 60% of patients with febrile illness receive medicine from the private sector [15–18]. In Uganda, the private sector provides approximately 50% of health services and this comprises of the private-not-for profit health facilities and purely private for profit facilities. The government has a public–private policy that supports the private sector through providing policy guidelines, support supervision and close collaboration in delivering some public health interventions like immunization, distribution of insecticide treated nets and family planning services [19].

Studies on referral in the private health sector in Uganda are scanty; thus a study was conducted with the main objective of assessing current referral practices among private providers for malaria, pneumonia, diarrhoea in order to inform the design of an intervention to improve referral of sick children.

## Methods

### Study setting

The study was conducted in Mukono, central Uganda. The total population of the district is 565,700 with an annual growth rate of 2.3% and consists of predominantly the Baganda ethnic group [20]. Most of the population, 88%, lives in the rural areas. Mukono district has a total of 6 hospitals, 4 health centres IV, 23 health centre III and 54 health centres II. A health centre II is the lowest level of health facility; it services a population of approximately 5000 and provides out-patient services. A health centre III services a population of approximately 50,000, has in-patient, maternity and laboratory services; while a health centre IV has an operating theatre and serves a population of about 100,000 people. A hospital has all the above services and serves a population of approximately 500,000 people. The private health sector in Mukono district comprises of private hospitals, private clinics, pharmacies and drug shops located mainly in urban areas and other built up trading centres. Hospitals, clinics and pharmacies are registered by professional councils while drug shops are registered by the national drug authority.

In the central region where Mukono district is located, 42.4% of children aged less than 5 years had fever, 9.4% had symptoms of acute respiratory tract infections, and 22.3% had diarrhoea in a previous household survey [2]. The district was selected based on previous studies that indicate that it has a good mix of both private and public facilities and has a high prevalence of childhood illnesses [11, 21, 22]. Thus, the study focuses on malaria, pneumonia and diarrhoea since these are the common childhood illnesses that may need referral.

### Study design

A survey was conducted from August to October 2014 in Mukono district, central Uganda. A list of all parishes (n = 84) in Mukono district was obtained from Uganda Bureau of Statistics. A parish is a geographically demarcated area with a population of approximately 5000 people. Study parishes were selected based on the following criteria: (i) contained a health centre II, the lowest public health facility where early treatment is sought; (ii) contained more than 200 households to ensure a sufficient number of patients visiting the facilities; and (iii) contained at least one registered drug shop, pharmacy or private clinic. The register of health facilities was used for selecting health facilities. Any private facility that was not in the district register was excluded. Clinics and drug shops were identified by their status of the registration and the minimum required facilities for each.

In total, 57 parishes that fulfilled that above criteria were selected; and a total of 241 private health facilities were surveyed (Table 2 shows the distribution of these facilities). Drugs shops represented 170/304 (55.9%) of the registered drug shops, private clinics, 59/69 (85.5%) and pharmacies, 12/25 (48%). Data was collected using a structured questionnaire targeting one provider who was found on duty in each selected private health facility and consented to the study (241 providers were interviewed).

**Table 1:** An example of the process of analysis

Meaning units	Codes	Themes
*“There are parents who think the child is going to die so they decide to ignore the referral” (FGD3 women)*	Caretakers losing hope when a child is referred	Barriers to referral at a household level
*“Most people will think that since the nurse has given me a referred form, it could be that it’s a complicated disease, the caretaker begins panicking” (FGD3 male)*		
*“These days everybody minds his own problems, you may go to a neighbour when you have a problem of referral or sickness but that person will not help you” (FGD women)*	Lack of support from community members	
*“But now people no longer have that helping heart so we need to teach them to support one another in case of illnesses; may be teach them through the church and any meetings held by the local council but people don’t like attending meetings” (KII private clinic)*		

### Participants

One staff in each private facility (drug shops, private clinics and pharmacies) who consented to the study was interviewed, using a semi-structured questionnaire to collect data on staff characteristics, including facility registration status, professional qualification of staff, previous training received, type of drugs stocked, availability of equipment/supplies/guidelines (by observation), knowledge of signs/symptoms and first-line treatment for uncomplicated malaria (was assessed using structured questions). These were supplemented by six focus group discussions (FGDs) and seven key informant interviews (KIIs).

Key informant interviews (KIIs) targeted community leaders serving on village local councils and members of village health teams (VHT) as well as health workers in private facilities and public health facilities on duty at the time the study was conducted. Two of the key informants were health workers in a government referral facility, two were health workers at private clinics who were found attending to patients at the time of the interview, two were members of village health teams and one was a member of the local council.

Focus Group Discussions targeted women and men who were taking care of children below five years in their homes. There were three FGDs for women which were held in three different villages. Two of the FGDs had six members each while the third had five members. There were three FGDs for men who were also from different villages. Two of the FGDs had six members each while the third had seven participants. The villages where male participants came from were different from those where female participants came from. Members of the FGDs were mobilized by the local council members in that village or the members of the village health team. The village health team works at village level to support health interventions in collaboration with the local council members. FGDs and KIs were conducted face-to-face through discussions in a convenient private place to ensure confidentiality and freedom of expression. The KIIs and FGDs guides focused on barriers to referral for sick children at both the community level and the health facility levels.

Referral was defined as sick children who were identified by a provider as ill and having one or more danger signs, and instructed to seek care from a higher level facility (usually health centre II, III, IV or hospital). This was measured by asking providers whether they had referred a sick child to weeks prior to the survey.

### Sample size calculations

Sample size was based on an estimated 40% of private health facilities that treat refer sick children. In order to estimate the proportion of referrals with a ±10% absolute precision, at a power of 80 and 5% level of significance (two-sided), and allowing for 10% non-participation, a minimum of 236 facilities were required.

### Data collection

Data were collected by six social scientists, well-versed with the local language Luganda, and English. Interviewers underwent refresher training for 5 days on qualitative research techniques, and study procedures. The questionnaires were pretested in the neighbouring district of Wakiso to assess the appropriateness of the questions. Revisions were done and the final questionnaires printed. Study team supervisors monitored and supervised data collection. Questionnaires with private sector health providers were administered in English, while KIIs were conducted in English for the health workers but in Luganda for the local council and VHT members. FGDs were conducted in Luganda and held separately for men and women. FGDs were tape-recorded after permission from participants had been sought and notes taken as well. Each FGD took between 30 and 50 min and ended when saturation was reached. Transcription was done immediately after conducting the interview or discussion. The interviews and FGDs were back-translated to English and ensuring that the original meaning from the respondents and participants was retained.

### Data analyses

Data was entered and cleaned using Microsoft Access 2007 (Microsoft Inc., Redmond, Washington) and analysed using STATA version 11.0 (STATA Corporation, College Station, Texas). Univariate analyses were performed to get proportions on key variables; and bivariate analyses were used to assess factors associated with referral sick children. Variables with a *p* value <0.05 were included in a logistic regression model to assess factors associated with referral of children while controlling for confounding for location of facility (urban/rural), type and level of facility.

Qualitative data was analysed using manifest content analysis [23]. Two research assistants analysed the data. Meaning units were picked and merged into codes. These codes were merged into themes. The analysis was iterative and at each stage, the investigators would meet, compare and agree on common themes. Sometimes, new issues would emerge and the text would be reviewed. The process of analysis is demonstrated in Table 1.

## Results

### Characteristic of private health facilities

A total of 241 private health care facilities were surveyed. The response rate was 98%; and the reason for non-response was absence of the provider in the selected health facility. The majority, 70.5% were drug shops; 24.5% were private clinics and 5.0% were pharmacies. Most of the facilities were busiest in the morning and evenings. Although 84.8% of private clinics had patient registers, only 36(21.2%) drug shops had patient registers. Most were selling anti-malarial drugs, amoxicillin, Zinc and ORS (Table 2).

**Table 2:** Characteristics of private health care facilities

Characteristics	Registered drug shops N = 170	Private clinics N = 59	Pharmacy N = 12
Type of facility	170 (70.5%)	59 (24.5)	12 (5.0%)
*Facility location*			
Urban	130 (76.5%)	53 (89.8%)	11 (91.7%)
Rural	40 (23.5%)	6 (10.2%)	1 (8.3%)
Facilities registered	125 (73.5%)	54 (91.5%)	12 (100%)
*Where facilities are registered*			
District (receipt seen)	29 (23.6%)	8 (14.9%)	0 (0.0%)
National drug authority (license seen)	93 (75.6%)	45 (83.3%)	12 (100%)
No response	1 (0.9%)	1 (1.9%)	0 (0.0%)
*What is the busiest time at this facility?*			
Morning (up to 12 p.m.)	46 (27.1%)	25 (42.4%)	9 (75.0%)
Afternoon (12 p.m.–5 p.m.)	10 (5.9%)	7 (11.9%)	0 (0.0%)
Evening (5 p.m.–7 p.m.)	19 (46.5%)	17 (28.8)	1 (8.3%)
Night (7 p.m.–12 a.m.)	20 (11.8%)	5 (8.5%)	2 (16.7%)
Facilities with a patient register	36 (21.2%)	50 (84.8%)	7 (58.3%)
Facilities selling anti-malaria drugs	170 (100%)	59 (100%)	7 (100%)
Chloroquine	21 (12.4%)	4 (6.8%)	5 (41.7%)
Fansidar (SP)	134 (78.8%)	52 (88.1%)	12 (100%)
Camoquine	11 (6.5%)	6 (10.2%)	1 (8.3%)
Quinine	141 (82.9%)	53 (89.8%)	11 (91.7%)
ACT	166 (97.7%)	57 (96.6%)	12 (100%)
Facilities selling amoxicillin	143 (89.4%)	58 (98.3%)	12 (100%)
Facilities selling zinc tablets	131 (77.1%)	50 (84.6%)	12 (100%)
Facilities selling ORS	164 (76.5%)	58 (98.3%)	12 (100%)
Facilities with stock control cards	27 (15.9%)	15 (25.4%)	7 (58.3%)
*Where facilities purchase drugs*			
From pharmacy	169 (99.4%)	59 (100%)	12 (100%)
Health units	0 (0.0)	0 (0.0)	0 (0.0)
Open markets	0 (0.0)	0 (0.0)	0 (0.0)

### Referral of sick children

Overall, 104/241 (43.2%) of the private health facilities reported that they had referred sick children to higher levels of care in the two weeks prior to the survey. Registered drug shops reported that they had referred 77/170 (45.3%) sick children and private clinics, 24/59 (40.7%). The facilities which referred sick children mainly referred them to health centres and hospitals. On average, private facilities see 5 sick children with fever per week, approximately 7 with cough and 3 with diarrhoea. Less than three children with severe illness are seen at the facilities per week (Table 3).

**Table 3:** Referral of sick children at private health care facilities

Children with pneumonia, diarrhoea and malaria seen at the private facilities	Registered drug shops N = 170	Private clinics N = 59	Pharmacies N = 12
Proportion of health facilities who reported that they referred sick children in the last 2 weeks	45.3% (77)	40.7% (24)	27 (3)
Mean number of sick children referred in the last 2 weeks (SD)	2 (3)	2 (1)	2 (1)
*Place where children were referred to*			
Drug shop	1 (1.3)	0 (0.0)	0 (0.0)
Private clinic	5 (6.5)	0 (0.0)	0 (0.0)
Health centre	43 (55.8)	11 (45.8)	1 (33.3)
Hospital	28 (33.4)	13 (54.2)	2 (66.7)
*Constraints encountered in referring children*			
Patients don’t comply	93 (54.7)	37 (62.7)	5 (41.7)
Referral facilities are too far	27 (15.9)	10 (17.0)	1 (8.3)
Patients don’t have money	93 (54.7)	37 (62.7)	5 (41.7)
There are no drugs at the referral facility	35 (20.6)	17 (28.8)	1 (8.3)

Factors that influence referral of sick children to a referral health facility were assessed. If the health worker was a registered nurse/midwife (OR 8.4); ever attended training workshops on management of malaria (OR 1.9); and the number of children with diarrhoea (OR 0.8) were factors that influenced whether a sick child was referred or not (Table 4).

**Table 4:** Factors that predict referral of sick children to higher referral facilities

Predictor variable (whether a child was referred or not)	Adjusted odds ratios	95% CI	*P*-value
Number of children with fever aged less than 5 years who visit the health facility per day	1.1	0.9–1.3	0.1
Number of children with cough aged less than 5 years who visit the health facility per day	1.1	0.9–1.2	0.07
Number of children with diarrhoea aged less than 5 years who visit the health facility per day	0.9	0.7–0.9	0.02
Gender of health worker (female)	0.6	0.3–1.5	0.3
Nursing assistant/aide	2.8	0.6–12.4	0.2
Enrolled nurse/midwife	1.5	0.3–7.0	0.6
Registered nurse/midwife	8.4	1.2–15.6	0.03
Clinical officer	2.7	0.4–18.3	0.9
Tertiary (certificate diploma)	1.2	0.6–2.2	0.6
Ever attended training workshops on malaria management	1.9	1.2–3.6	0.04
Ever attended training workshops on pneumonia management	0.9	0.3–2.4	0.8
Ever attended training workshops on diarrhoea management	0.7	0.3–1.4	0.3
Whether thermometer is available at health facility	1.1	0.3–4.0	0.8
Whether functioning microscope is available at health facility	1.2	0.5–2.8	0.7
Whether malaria rapid diagnostic test is available at health facility	0.9	0.5–1.7	0.7
Whether a copy of malaria treatment guidelines is available at health facility	1.3	0.6–2.7	0.5

### Characteristics of referral facilities

Of the referral facilities 55.8% were HCII followed by HCIII (30.8%) and a few were HCIV and hospitals. At referral facilities 64.2% of staff were trained in malaria, but <30% had training in pneumonia and diarrhoea case management, despite this, the majority had malaria treatment and IMCI guidelines (Table 5).

**Table 5:** Characteristics of higher level referral facilities where children were referred

Characteristics	N = 53
*Level of facility where children were referred*	
Hospital	2 (3.9)
HC IV	5 (9.6)
HC III	16 (30.8)
HC II	29 (55.8)
*Training in case management of childhood illnesses*	
Malaria management	34 (64.2)
Pneumonia	11 (20.8)
Diarrhoea	16 (30.8)
*Availability of treatment guidelines*	
Malaria	38 (71.7)
IMCI	35 (66.0)
Average number of children sick seen in the last 1 week (mean, range)	3 (2–5)
Average number of children sick referred in the last 1 week (mean, range)	3 (1–7)
Knowledge that Coartem (ACT) is the first-line anti-malarial drug for uncomplicated malaria among children	49 (92.5)
Knowledge that Amoxicillin is the first-line treatment for pneumonia in children	26 (49.1)
Knowledge that ORS and Zinc are the first-line treatment for diarrhoea in children	50 (94.3)
Knowledge that convulsions, high temperature, vomiting and unconsciousness are severe symptoms/signs for malaria	43 (81.1)
Knowledge that rapid breathing, difficulty in breathing, cough, chest in-drawing are severe symptoms/signs for pneumonia	49 (92.5)
Knowledge that dehydration (dry lips, elastic skin, dry skin); sunken eyes, watery stools and general body weakness are severe symptoms/signs for diarrhoea	48 (90.6)

### Barriers to referral of sick children at health facilities

#### High costs of services and drug stock outs at referral facilities

A reason for people not taking up referral was the risk of high costs at referral facilities for drugs and utilities. Lack of equipment and drugs at the referral facilities was also a problem.

“They may refer you to a government facility, but you will pay for the medicines. When you go to a clinic ok, they will ask for money like 10,000/= but in a hospital they will even charge you for the bed you have used and at times you may not have the money to pay, so sometimes you say that someone who has remained at home is better” (FGD1 women)

“We don’t take patients there. We take them to clinics where we are used to because they can treat on credit and you pay later. There are no drugs [in health facilities]. You can’t even tell anyone that you took a child to the higher level facility. They will laugh and say you didn’t love that child” (FGD1 male)

#### Low expectations of treatment at referral facilities

It was widely mentioned that the service at referral facilities was poor, there were long waiting hours, and drugs were often out of stock. The motivation and commitment of health workers was also repeatedly questioned. Participants indicated that receiving treatment at the referral facilities could depend on ability to pay the health workers, whilst the possibility that a government-employed health worker may also ran a drug shop or private clinic further undermined confidence in their integrity and motivation to provide care.

“Then after being referred we may go so early to the health facility but they delay to give us treatment so this needs to be improved. You may get there at 7:00 a.m. but get treatment at 1:00 p.m. and in case the patient is very sick he or she may die so they need to take better care of us and reduce waiting time to at least 1 h. And at 1:00 p.m. this person is told to go and buy drugs so there is no help in the health facility” (FGD2 male)

“The government facilities have drugs but there is a lot of corruption in these government facilities, if you go to the government facility when you have money they will give you the drugs but if you are not paying they will only prescribe for you to go and buy” (FGD2 women)

Private providers and the village leaders indicated that people refuse to take up referrals because of not expecting drugs at the referral facilities but also the long waiting time they have to spend there before they get treatment.

“Those who fail to take up the referrals do so because they have to look for money for transport, then others don’t want to go to government health facilities because there are no drugs, then others say the lines are so long so they have to wait for so many hours before they get treatment and there is congestion” (KII private clinic)

However, one of the respondents from the referral facility indicated that the long lines should not hinder people from seeking care from the government facilities.

“There is a myth with people saying, ‘I will not go to the health centre because of the long lines and there is no care from the health workers, there are stock-outs and I will need to buy drugs any way’. So they stay home and don’t think that their children need better medication so they need to be sensitized that we health workers are there to serve them and since they are many, drugs may go out of stock but we are doing a great job so they should come for medication” (KII government referral facility)

The capacity of some (lower level) health facilities was not much better than that of the private clinics hence were not used as referral facilities.

“When I go to the clinic and am told that the patient needs a referral I make sure that I go where I have been told to go but the challenge is that from the clinic [health centre level II] cannot handle the situation so you have to go to a mission hospital and if you have no money you just go back home” (FGD1 male)

#### Long distances to referral facilities

One of the issues raised by FGD participants was that the health facilities were far away and the people needed transport, not often available and health workers not being at the facility on night duty also needed transport to go to the clinic.

“The major constraint to take referral at Kiyagi is lack of transport, then when you get there at night it is 11:00 p.m. the patient is so weak and you have to call the health worker who stays in Nakifuma, and she says I need transport” (FGD1 male).

“One time some people from Nakiduduma brought a child in the morning and after examining the child, she was anaemic so I told them that the child was anaemic and I advised them to take the child to a hospital either Kayunga, Nagalama or Nakifuma. They started complaining that they had no money but they got someone to lend them some money for transport but couldn’t afford using a special hire so they had to wait for the taxi to get full with passengers but while at the hospital as they were still in the queue the baby died. So at around 11:00 they told me that the child had died. This implies that they took so long on the way because the taxis need money so cannot move before getting the right number of people they need, so the child died” (KII private clinic)

### Barriers to referral seen from the caregiver’s perspective

One of the perceived barriers to referral was the suspicion by caregivers that private providers were focused on making profit. The participants in all FGDs characterized health workers in private health facilities as having love for money that could prevent them from referring children either by refusing to refer and continue to profit by selling medicines to the client or by refusing to refer them until bills they may have accumulated at the facility were paid.

“Ok sometimes you may take your child to a clinic, they first give some treatment and after seeing no improvement, they refer to either Mulago or Nagalama yet you have to first clear her bill, so you will have no money and eventually fail to take your child to where you have been referred. Yet you cannot leave without paying her” (FGD3 women)

#### Lack of confidence in private providers

The attitude of caretakers with respect to the competence of private providers was identified as a barrier to the uptake of referral. On being referred, some caretakers often thought that the reason that health workers referred their children was that they were not qualified to handle the illness. Instead they would go to another nearby clinic. This is highlighted in one of the FGD with women below:

“For me when I go to a clinic with a small complication and a health worker refers me, I regard that person as someone who is not trained, some people try life in different ways, someone will work in a clinic when he/she is not a health worker” (FGD2 women).

The private providers were aware of the above attitudes by caregivers. Private providers said that some caretakers do not mention that they have been to another facility.

“So when I refuse to give any medication he goes to another clinic where the health worker may treat as he wishes or the caretaker may hide the child then tells the other health worker anything so that he gets some medication so he may be given drugs for Uganda shillings 500 and at times the child dies” (KII private clinic).

Awareness by the provider that making a referral might expose a lack of competence, and ultimately undermine their reputation, was also given as a factor hindering referral of children.

“They don’t give referral letters because of lack of confidence to state the diagnosis, whether the treatment given is the correct one. So she will imagine that making a referral letter puts her in problems so she will verbally tell you go to a hospital” (FGD3 male)

### Barriers to referral at a household level

#### Home treatment as a barrier to taking up referral

Home treatment was perceived as another barrier to taking up referral advice as highlighted in following quotes:

“So when my child falls sick and I am referred, I will treat the baby myself, I will buy some Panadol and chloroquine and give the child, and by chance the child may recover, if the chance is bad the child will die” (FGD2 women)

“Some of us parents may be reluctant and say “aah the child will be fine” so they give herbs” (FGD1 male)

#### Negligence by caretakers

Negligence on the part of the caretakers was highlighted as one of the reasons why they do not take up referral. This was mentioned in both female and male FGDs.

“May be about the other issue, there are some parents who are taking care of orphans whose parents died of HIV/AIDS, but if such a child falls sick they first take him/her to a clinic like the one in Kisowera that we’ve told you about, so in case of a referral, they will just say that ‘’eeeh am not the one who told your parents to die of HIV/AIDS’’, so they will ignore the baby” (FGD 3 women)

#### Lack of awareness on the importance of referral

Lack of awareness on the importance of referral was highlighted as a reason why people do not take up referral.

“People are not aware; we still need some sensitization for people to know the dangers of not taking up referral. Sometimes the sensitizations are there but people don’t always go to the gathering whenever they are called upon” (KII Local Council member)

Participants indicated that another reason why people do not take up referral advice is fear of blood tests.

“We have noticed that people decline to take up referral because they fear blood tests. So some parents fear that they will be tested for HIV (FGD3 male).

#### Loss of hope by caretakers

In contrast, some caretakers lose hope when confronted with referral. They perceive the child to be suffering from a very serious illness and that chance of survival is very low and therefore they lose hope.

“There are parents who think the child is going to die so they decide to ignore the referral” (FGD3 women)

“Most people will think that since the nurse has given me a referred form, it could be that it’s a complicated disease, the caretaker begins panicking” (FGD3 male)

#### Decision making at a household level

Female caregivers said that sometimes they disagree with their husbands on whether to take up the referral or not such discussions delays action. Sometimes these disagreements were due to a personal attachment to a particular health worker at the clinic and a preference that this health worker be the one to treat the child.

“You may go there and they give you some drugs and the child may fail to improve and the husband may insist that you treat the child from that particular clinic; and the woman will say we must go to a big facility, and you will struggle with the husband…. so sometimes it is the husbands who influence their wives not to honour these referrals and by the time you come to an agreement the condition of the child will have worsened” (FGD1 women)

“Sometimes a man may have money but the moment this man has a friend who is a health worker in one of the clinics around he will always want that health worker to be the one to treat his children however bad the condition is” (FGD1 women)

In FGDs it was highlighted that men can shun their responsibilities of looking after children. This can make it difficult for women to take up referral without the support of the men.

“Men don’t care about their children. It is only women who are caring about their children, not only treatment but also paying school fees …it is women who are struggling so men have not done their part, they have thrown out their responsibilities” (FGD2 women).

“And another thing is that some mothers are abandoned by the husbands, so they think of how they will manage the lonely situation in the hospital and end up with fear to go to the hospital and opt to keep seeking care from the clinics. At home she has other children to take care of, so when a child is sick, there is no one at home to take care of the other children, so she fears to go to the hospital when referred”(FGD3 male).

#### Financial barriers

Financial constraints were perceived as a main barrier to taking up referral advice as iterated in quotations below.

“The reason why people do that is because of little money or lack of enough money to take them where they have been referred. Someone will imagine the source of money to take the child to where it has been referred and no hope to get it, so it’s not that you don’t want but because you cannot afford. But if you can afford and you have the money, you take a child where she has been referred” (FGD3 women).

“It is money because like in Nagalama [private] hospital the health workers are available all the time and they have the drugs so it is money for transport and upkeep while in hospital that is lacking that hinders them from taking up these referrals but they would have respected them if they had had money” (KII village health team member).

#### Inadequate community support

Participants noted that community cohesion had been greatly reduced and people were not supporting each other in times of need. This made it difficult for people to get support for referrals.

“These days everybody minds his own problems, you may go to a neighbour when you have a problem of referral or sickness but that person will not help you” (FGD women)

“But now people no longer have that helping heart so we need to teach them to support one another in case of illnesses; may be teach them through the church and any meetings held by the local council but people don’t like attending meetings” (KII private clinic).

## Discussion

The results show that referral of sick children from private facilities had many constraints. The factors that influenced referral of a sick child were the level of qualification of the health worker, the availability of IMCI guidelines and the number of sick children seen at the facility. The qualitative data also shows that one of the reasons for the poorly functioning referral chain is that people have no trust in the government services. Also, they stated that they have to pay informal fees there, without knowing if they will actually get the treatment desired by them (such as drugs). Data further shows that the staff in the public facilities are interested in their own private practices and therefore do not perform up to standard in their public role. These are some of the reasons why the referral system is performing poorly.

Other barriers perceived to affect referral of sick children include inadequate finances at a household level, caretakers not appreciating the importance of referral, gender issues and decision making, the perception by private providers that referral leads to loss of prestige and profit; poor quality of care at referral facilities (drug stock-outs, negative attitudes of health workers, lack of adequate equipment and supplies); long distances to referral facilities.

The above findings are consistent with previous research on referral practices in Uganda and elsewhere. For example, previous studies have shown that several factors influenced poor referral practices and uptake of referral, including long distances to health facilities, high costs involved, poor attitudes of health workers, lack of drugs at health facilities and lack of involvement of fathers in the referral process [3, 7, 11, 17, 18, 20–22].

In a recent study in drug shops in Uganda, it was shown that referral of patients was inappropriate and the main constraints were lack of acceptability of the referral form written in the local language by the health workers at referral facilities; and mistrust between drug shops and health workers led to poor acceptability of referral [17, 18].

The present results show that the qualification of providers at registered health facilities and training in malaria management were important in referring children at these facilities. The implication of these findings is that the national drug authority and the allied health professional council could use this evidence to review the minimum qualification of a registered nurse/midwife to register a drug shop and a private clinic. The Ministry of Health with support of the Global Fund and other multi-lateral partners could also use this evidence to discuss ways of training of private providers in management of common childhood illnesses.

Interventions to improve referral are needed to address constraints at community level to address factors like inadequate finances and how to design saving schemes at a community level; creating awareness for caretakers to appreciate the importance of referral; involving men and the “significant others” at a family level to improve gender-related negotiations and decision-making related to health-seeking (Table 6).

**Table 6:** .

What is already known about this subject? In Uganda studies have shown that referral of sick children seen in public health facilities to higher levels of care is poor and faces several constraints. Studies focusing on the private health sector are scanty	
What does this study add? Data presented in this study shows that referral of sick children at private health facilities faces many constraints ranging from caregivers not appreciating the importance of referral, gender-related decision making and negotiations at household level, poor quality of care at referral facilities and inadequate finances at household level	
How might this impact on clinical practice? Addressing the identified referral constraints will reduce morbidity and mortality among children seen in the private health sector in Uganda	

One important finding is the poor quality of care at referral facilities namely drug stock-outs, negative attitudes of health workers, lack of adequate equipment and supplies. To improve quality of care these issues need to be addressed in a sustainable manner. One innovative intervention to address this would be to engage the public in discussions that would enable health workers to understand the issues that discourage patients from seeking care at referral facilities. Engagements involving the community users, the local leaders and health workers could probably change the attitude of health workers. This is recommended this for further research.

The limitations of this study are that it was not possible to estimate the accuracy of referrals from private facilities due to poor record keeping as previously noted [22]. Similarly, private providers were asked questions whether they referred a sick child in the last 2 weeks but we were not able to investigate whether the referral advice was followed or not. This however required a household survey that was beyond the scope of this study and it is recommended for further research. It was also not possible to observe referral practices at these facilities and thus the results are limited to self-report from private providers.

Nonetheless, the survey (supplemented by qualitative findings) covered a large number of private health facilities in Mukono district that has many similarities to most districts in the country in terms of private–public health facility mix and a similar pattern of childhood diseases. Thus, these results could be generalized to most parts of Uganda.

The immediate policy implications of these findings is the Ministry of Health to consider disseminating IMCI guidelines to the provide providers; and provide support supervision to ensure their proper use. There is also need to discuss these results with district leaders, the national drug authority and the professional councils that regulate private practice in Uganda and identify solutions to the constraints and barriers to effective treatment and referral of sick children.

Following this study, an intervention was designed, a cluster randomized study (NCT02450630; registered with ClinicalTrials.gov; on 20th May 2015); currently under implementation to assess the effect of strengthening treatment and referral of sick children from the private health sector in Uganda. The key elements of the intervention are: community awareness on referral, training private providers to use RDTs and ICCM algorithms to treat and refer sick children; regular meetings between the private and public sector and proper documentation of referral practices. It is hoped that the evaluation of this intervention will provide useful lessons to improve referral of sick children from the private sector.

In conclusion, the results show that referral of sick children at private health facilities faces many challenges. Thus interventions to increase referral of sick children are urgently needed.

## References

[1] Uganda Bureaux of Statistics. Uganda Population and Housing census. Kampala. 2014.

[2] Uganda Bureaux of Statistics. Uganda Demographic and health Survey. Kampala. 2011.

[3] Achan J, Tibenderana J, Kyabayinze D, Mawejje H, Mugizi R, Mpeka B (2011). Case management of severe malaria–a forgotten practice: experiences from health facilities in Uganda. PLoS ONE.

[4] Font F, Quinto L, Masanja H, Nathan R, Ascaso C, Menendez C (2002). Paediatric referrals in rural Tanzania: the Kilombero District Study—a case series. BMC Int Health Hum Rights..

[5] Oryema-Lalobo M (2009). Community health seeking practices for the management of malaria of the under-five in Bugiri District, Uganda. East Afr J Public Health..

[6] Källander K, Tomson G, Nsungwa-Sabiiti J, Senyonjo Y, Pariyo G, Peterson S (2006). Community referral in home management of malaria in western Uganda: a case series study. BMC Int Health Hum Rights..

[7] Peterson S, Nsungwa-Sabiiti J, Were W, Nsabagasani X, Magumba G, Nambooze J (2004). Coping with paediatric referral–Ugandan parents’ experience. Lancet.

[8] Samuelsen H, Tersbøl BP, Mbuyita SS (2013). Do health systems delay the treatment of poor children? A qualitative study of child deaths in rural Tanzania. BMC Health Serv Res..

[9] Mbonye AK (2003). Prevalence of childhood illnesses and care-seeking practices in rural Uganda. Scientific World J..

[10] Sserwanga A, Sears D, Kapella BK (2015). Anti-malarial prescription practices among children admitted to six public hospitals in Uganda from 2011 to 2013. Malar J..

[11] Mbonye AK, Lal S, Cundill B, Hansen KS, Clarke S, Magnussen P (2013). Treatment of fevers prior to introducing rapid diagnostic tests for malaria in registered drug shops in Uganda. Malar J..

[12] Källander K, Hildenwall H, Waiswa P, Galiwango E, Peterson S, Pariyo G (2008). Delayed care seeking for fatal pneumonia in children aged under five years in Uganda: a case-series study. Bull World Health Organ.

[13] Font F, Quinto L, Masanja H, Nathan R, Ascaso C, Menendez C (2002). Paediatric referrals in rural Tanzania: the Kilombero District Study—a case series. BMC Int Health Hum Rights..

[14] Birungi H, Mugisha F, Nsabagasani X, Okuonzi S, Jeppsson A (2001). The policy on public-private mix in the Ugandan health sector: catching up with reality. Health Policy Plan..

[15] Nsungwa-Sabiiti J, Peterson S, Pariyo G, Ogwal-Okeng J, Petzold MG, Tomson G (2007). Home-based management of fever and malaria treatment practices in Uganda. Trans R Soc Trop Med Hyg.

[16] Mbonye AK, Ndyomugyenyi R, Turinde A, Magnussen P, Clarke SE, Chandler C (2010). The feasibility of introducing rapid diagnostic tests for malaria in drug shops in Uganda. Malar J..

[17] Kozuki N, Guenther T, Vaz L, Moran A, Soofi SB, Kayemba CN (2015). A systematic review of community-to-facility neonatal referral completion rates in Africa and Asia. BMC Public Health..

[18] van der Geest S (1987). Self-care and the informal sale of drugs in South Cameroon. Soc Sci Med.

[19] Ministry of Health. Health Sector Strategic Plan III. Ministry of Health. Kampala. 2010.

[20] Chandler CI, Hall-Clifford R, Asaph T, Pascal M, Clarke S, Mbonye AK (2011). Introducing malaria rapid diagnostic tests at registered private outlets in Uganda: limitations of diagnostic testing in the reality of diagnosis. Soc Sci Med.

[21] Hutchinson E. Evaluation of the referral system implemented in registered drug shops in Uganda. Final Evaluation Report. 2012.

[22] Hutchinson E, Chandler C, Clarke S, Lal S, Magnussen P, Kayendeke M (2015). ‘It puts life in us and we feel big’: shifts in the local health care system during the introduction of rapid diagnostic tests for malaria into drug shops in Uganda. Crit Public Health..

[23] Hsieh HF, Shannon SE (2005). Three approaches to qualitative content analysis. Qual Health Res.

